# Treatment effect heterogeneity in the head start impact study: A systematic review of study characteristics and findings

**DOI:** 10.1016/j.ssmph.2021.100916

**Published:** 2021-09-08

**Authors:** Sun Yeop Lee, Rockli Kim, Justin Rodgers, S.V. Subramanian

**Affiliations:** a Department of Epidemiology, Harvard T.H. Chan School of Public Health Boston, MA, USA; b Interdisciplinary Program in Precision Public Health, Department of Public Health Sciences, Graduate School of Korea University, Seoul, Republic of Korea; c Division of Health Policy and Management, College of Health Science, Korea University, Seoul, Republic of Korea; d Harvard Center for Population & Development Studies, Cambridge, MA, USA; e Department of Social and Behavioral Sciences, Harvard T.H. Chan School of Public Health, Boston, MA, USA

**Keywords:** Head start, Head start impact study, Child development, Childhood intervention, Treatment effect heterogeneity, Heterogeneous effect

## Abstract

There have been consistent efforts to assess treatment effect heterogeneity (TEH) of Head Start using the data from the Head Start Impact Study (HSIS), a randomized controlled trial of a federally funded child development program for a nationally representative sample of low-income parents and their 3- and 4-year-old children in the United States. Including 28 studies on TEH of Head Start, this review found that multiple high-risk subgroups (e.g., children with lower cognitive abilities, Spanish-speaking dual language learners) experienced larger gains across a range of developmental and parental outcomes, but mixed results for several subgroups. Most studies focused on subgroup analyses, cognitive and social-emotional outcomes, and short-term effects. Further studies on distributional effects, health and parental outcomes, and long-term effects are warranted. Finally, suggestions for future research on TEH of Head Start are discussed, which are applicable to other child development programs and policy evaluations.

## 1 Introduction

### 1.1 Head start

Initiated in 1965, Head Start is one of the largest and the only federally funded early childhood developmental program in the United States that assists low-income parents and their children. Since its inception, the program has served more than 37 million children and their families, playing an important role in improving welfare and population health, as well as alleviating social inequalities. Between fiscal year 2016 and 2019, the program gained $890 million increase in funding, and in 2020, the funding was set at $10.61 billion. The program continues receiving bipartisan support (*Head Start & Early Head Start*, 2020).

Based on a “whole child” model, the program has four components: education, health, social services, and parent involvement. It promotes academic success, improved management of health needs, and positive social and behavioral development by providing various services including early childhood education; medical, dental, and mental healthcare; nutritional services; and parenting support. The program strategically engages parents and key family members into services to support family well-being and promote children's growth and development through family-centered approaches. Most Head Start centers are run by non-profit organizations, schools, and community agencies, and they all follow the Head Start Program Performance Standards to ensure provision of high-quality early education and child development services (Puma et al., 2010).

### 1.2 Head Start Impact Study and treatment effect heterogeneity

The Head Start Impact Study (HSIS), a randomized controlled trial of Head Start, was conducted in 2002–2008 on nationally representative cohorts of 3- and 4-year-old Head Start applicants to assess the overall effectiveness of Head Start and for whom it is effective. The study participants were selected by multi-stage sampling design and consisted of the Head Start applicants at 383 Head Start centers nested in 84 program agencies across 23 states. The children were randomly assigned to either a treatment group with access to one year of Head Start or a control group without access to Head Start. The children and their parents were followed up until the end of their third grade year to assess how one year of Head Start at the age 3 or 4 affects children's cognitive, social-emotional, and health development and parenting practices. More details on the HSIS have been described in the official reports of the HSIS (the HSIS reports, from now on) (Puma et al., 2010, 2012).

The HSIS reports have concluded that the Head Start had beneficial impacts on multiple cognitive, social-emotional, health, and parental outcomes for the study participants at the end of Head Start, although most of these impacts had faded according to follow-ups in the subsequent years, pre-kindergarten to third grade. However, subgroup analyses in the HSIS reports told a different story; some subgroups defined by participants’ pre-treatment, or baseline, characteristics (e.g., dual-language learners, children with lower baseline cognitive skills) experienced much greater beneficial impacts that were also long-lasting, and for a few outcomes, sustained through third grade. Subgroups with different baseline characteristics can experience heterogeneous effects because they may respond differently to the treatment or differ in the outcomes they would have achieved without the treatment (i.e., counterfactual outcomes) (Kravitz et al., 2004). For example, in terms of receptive vocabulary, Head Start may benefit Spanish-speaking dual language learners more than English-speaking children because those who are already fluent in English would have scored high on receptive vocabulary in the absence of the extra support from Head Start. Alternatively, English-speaking children may benefit more because the Head Start curriculum may be more suitable for those with greater exposure to English. Other sources of treatment effect heterogeneity (TEH) may present in Head Start (Plewis, 2002). Its effectiveness may vary by states or regions depending on the different educational standards or regulations. Also, some Head Start centers may be more effective than others because each center independently modifies the nationally guided practices to meet specific community needs.

The multidimensional nature of Head Start services and the wide range of its target recipients make it difficult to clearly lay out who is benefitting how much from Head Start. Each child is expected to respond distinctively to such child development program, considering their unique set of biological predispositions, and demographic and sociological attributes. Some children may experience greater or smaller benefits than the treatment effect on average, or average treatment effect (ATE). Understanding such TEH is particularly relevant for early childhood developmental programs because these programs are major parts of children's experiences during the critical periods of development (Halfon & Hochstein, 2002). Scientific advances in understanding of child development, such as neuroplasticity and the critical periods, call for better-targeted interventions that are based on causal mechanisms and TEH (Shonkoff, 2017). A careful attention to TEH during the design and evaluation phase of these interventions is of vital importance, and the inability to meet the heterogeneous needs of the population would make the interventions inefficient and susceptible to leaving certain groups behind (Kravitz et al., 2004; Subramanian et al., 2018).

Therefore, we provide a comprehensive review summarizing all TEH studies using the HSIS data. The large number of studies published since its last official summary report in 2012 warrants a summary of their own, and in addition, the HSIS data would be the most appropriate source to examine TEH of Head Start due to its RCT design and the national representativeness of the sample. Our three specific aims are to 1) identify subgroups of children for whom there was strong evidence for beneficial impacts or conflicting findings, 2) illuminate deficient areas of research in terms of study outcomes and follow-up periods, and 3) summarize and examine the trend in the analytical approaches to assess TEH. To accomplish the aims laid out above in a systematic review, we first describe characteristics of the studies, including the outcomes of interest, moderators, targeted cohorts, assessment years, and analytical methods. Next, we summarize the findings on the heterogeneous Head Start effects and identify common trends. We also report quantitative summaries of the overall trend in statistically significant findings. Finally, we offer explanations for the trends observed in the treatment effect findings and suggest future directions on investigating the Head Start effects, which are applicable to the broader field of child development program and policy evaluation.

## 2 Methods

### 2.1 Search strategy

To identify relevant studies on TEH of Head Start using the HSIS data, nine electronic databases were utilized: PsycInfo, PsycArticles, Econlit, Education Resources Information Center (ERIC), PubMed, Embase, Web of Science, Academic Search Alumni Edition, and Academic Search Premier. A single keyword, “head start impact study”, was used in the systematic search to ensure all relevant studies were captured. References of these studies were also reviewed to further identify additional relevant studies.

### 2.2 Inclusion/exclusion criteria

To be included in the review, studies needed to meet following criteria: 1) use of the HSIS data; 2) analysis of TEH; 3) evaluation of the Head Start effect on cognitive, social-emotional, health, or parental outcomes, which are the main outcomes of interest listed in the HSIS reports. Empirical studies published in peer-reviewed journals and institutional reports were included. Dissertations and conference proceedings were checked whether they were later published in peer-reviewed journals, and their published versions were included. If there were no published versions available, dissertations were included but conference proceedings were excluded due to lack of necessary information for the review.

### 2.3 Study selection

Two reviewers, SL and RK, determined inclusion and exclusion of the studies. After the literature search on the electronic databases, the identified studies were imported to a systematic review software (Covidence systematic review software). SL removed duplicates identified by Covidence. Then, SL screened titles and abstracts to exclude irrelevant studies, and RK double-checked the included studies and excluded studies for their appropriateness. Lastly, a full text of each study was reviewed independently by SL and RK to ensure that it met the inclusion/exclusion criteria.

### 2.4 Data extraction

The following information was extracted from the studies included in the review: 1) bibliographic information on each study, which included author(s), title, and year of publication; 2) study characteristics which included outcomes (cognitive, social-emotional, health, or parental), targeted cohorts (3-year-old, 4-year-old, or combined cohort), assessment years, moderators (child, household/parental, neighborhood, or center characteristics, or child care types), parameters of interest (average treatment effect (ATE), quantile treatment effect (QTE), variance, or individual treatment effect), and analytical methods. The outcomes were categorized as they were in the HSIS reports (see page 25 of the 2012 HSIS report for their definitions) (Puma et al., 2012). When possible, composite measures of outcomes were separated into individual outcomes to be transparent on what is being investigated. The combined cohort is a pooled cohort of the 3- and 4-year-old cohorts, and they were pooled by the number of years since Head Start assignment (Year 1 to Year 3) or academic year (age 4 to 3rd grade) (Table A4).

### 2.5 Analytic approach

A standard meta-analysis was not performed as our review aimed to emphasize heterogeneity in treatment effects and included studies on a large number of different outcomes and moderators at different assessment years. Moreover, since we focused on studies using the HSIS data, if more than one study had analyzed an outcome at the same assessment year using the same moderator, any difference in estimates would be due to different analytic approaches rather than the presence of true TEH. Instead, our review focused on quantitatively and qualitatively synthesizing the studies to describe the trends in study characteristics and study findings.

We first summarized the frequency of study characteristics among all included studies by presenting proportions of studies with each study characteristic. Proportions of each outcome within an outcome category (Table A2), each moderator within a moderator category (Table A3), and each assessment year by cohorts (Table A4) are also reported. In addition, we also report the number of outcomes in the HSIS reports for which TEH was under- or uninvestigated.

Next, to highlight the frequently analyzed findings in detail, qualitative summaries by moderators are provided in four ways. First, if the subgroups defined by the moderators were analyzed in at least 3 studies, they were organized into those that had consistently beneficial effects across multiple outcomes and those that had mixed results across outcomes, and then their subgroup or interaction analysis findings were summarized. Second, study findings on distributional effects were summarized. Distributional effects are defined as the effect of an intervention on the outcome distribution. While an ATE represents whether an intervention increased or decreased the outcome on average, a distributional effect describes how the distribution of an outcome changed after the intervention. The outcome distribution can widen, shrink, or shift, depending on the effect of an intervention on individuals. Third, a summary was provided for the studies that decomposed and quantified the proportions of explained and unexplained TEH.

For a quantitative summary, a contingency table was constructed to list proportions of statistically significant findings at the 0.05 level of significance by the intersections of outcome and moderator categories to summarize the overall trends in findings. In addition, proportions of statistically significant findings were also reported by assessment years to compare trends over time, summarizing short- and long-term effects. When studies utilized multiple analytical methods or models for the same hypotheses (e.g., for sensitivity analyses), primary methods and models were included in the contingency tables.

## 3 Results

**Fig. 1:**
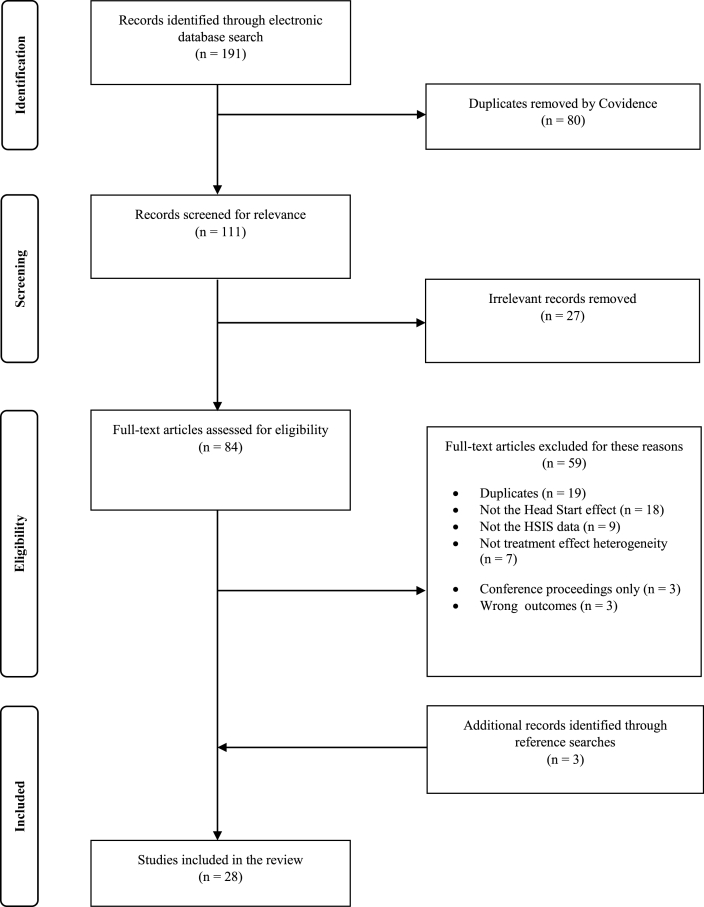
Flow diagram for study selection.

Among 191 studies identified by the database search, 80 duplicates were identified by Covidence and removed by the reviewers. From the remaining 111 studies, additional 19 duplicates in alternative scholarly forms (e.g., abstracts) were removed, and 27 studies were considered irrelevant from screening their titles and abstracts. A full-text assessment excluded studies for following reasons: not assessing the Head Start effect (n = 18), not using the HSIS data (n = 9), not assessing TEH (n = 6), only available in the form of conference proceeding (n = 3), and assessing the Head Start effect on child care experiences, rather than child developmental and parental outcomes (n = 3). Note that child care experiences were not the ultimate goal of Head Start, but rather a mediator through which Head Start intended to improve participants’ cognitive, social-emotional, health, or parental outcomes. Adding three more studies from reference searches, the final number of studies included in the review was 28 (Fig. 1).

### 3.1 Study characteristics

The detailed characteristics of all included studies are presented in Table A1. In Table 1, categorization of the studies is not mutually exclusive because most studies overlapped in their characteristics. Twelve studies (43%) included more than one outcome category, 12 studies (43%) investigated more than one moderator category, and six studies (21%) analyzed more than one cohort. Consequently, the sum of all percentages within a category often exceed 100 percent.

**Table 1:** Frequency of study characteristics.

Study Characteristics	Category	# of studies	Proportion among all studies (%)^a^
Outcome	- Cognitive	20	71
	- Social-emotional	13	46
	- Health	2	7
	- Parental	7	25
			
Targeted cohort	- 3-year-old	8	29
	- 4-year-old	6	21
	- Combined	23	82
Moderator	- Child characteristics	14	50
	- Household/parental characteristics	13	46
	- Child care type	8	29
	- Center characteristics	4	14
	- Neighborhood characteristics	2	7
Parameter of interest	- Average treatment effect	26	93
	- Quantile treatment effect	2	7
	- Variance	2	7
	- Individual treatment effect	2	7
**Total # of studies**		**28**	

a Sum of the proportions may exceed 100 percent because categorization of the studies is not mutually exclusive.

#### 3.1.1 Outcomes

Most studies focused on cognitive (71%; n = 20) and social-emotional outcomes (46%; n = 13), while a small portion assessed parental outcomes (25%; n = 7). Only two studies (7%) assessed health outcomes (Table 1).

Among studies on cognitive outcomes, the majority focused on Applied Problems (80%) which measures skills for analyzing and solving math problems, Letter-Word Identification (75%), and PPVT (Peabody Picture Vocabulary Test) (70%) which measures receptive vocabulary as the main outcomes. Spelling (35%), Oral Comprehension (30%), and Quantitative Concept (15%) were examined less frequently. Among the 26 cognitive outcomes from the HSIS report, seven outcomes, such as Color Identification and Word Attack, were studied only once, while 13 outcomes, such as Passage Comprehension, Calculation, and teacher- and parent-reported school performance, were never studied (Table A2).

Studies on social-emotional outcomes analyzed parent-reported outcomes (85%) and teacher-reported outcomes (46%). The studies mainly used parent-reported aggressive and hyperactive behaviors (77%), and parent-reported social skills and positive approaches to learning (62%) as outcomes. Ten out of 17 teacher-reported outcomes, such as measures from the Strengths and Difficulties questionnaire, and all four child-reported outcomes, which assessed child's academic and social skills, have not been analyzed.

Two studies examined health outcomes, and they both analyzed the children with non-parental care at baseline. Pratt et al. (2015) used information on dental care receipt, hearing screening, vision screening, and access to regular medical check-up to evaluate the effect of Head Start on health service utilization. Lee (2020) assessed the Head Start effect on dental care receipt and medical care for an injury in the last month. Three out of five official-report health outcomes (i.e., access to health insurance, parent-reported child's health, and the need for ongoing medical care) have not been examined.

Studies on parental outcomes examined how Head Start affects the frequency of book reading for the child by parents (60%), physical discipline, and family cultural enrichment activities (40%). Ten out of 13 official-report parental outcomes, such as use of time out, parent participation in school, time spent with child, have not been analyzed.

#### 3.1.2 Moderators

TEH of Head Start was explored most frequently by child characteristics measured at baseline (Table 1; 50%; n = 14). Forty-three percent of these studies used children's baseline cognitive skills, 36 percent used children's primary language and special needs status, and 29 percent used gender as potential moderators (Table A3).

Almost a half (46%; n = 13) explored TEH by household/parental characteristics. Forty-six percent of these used race/ethnicity as a moderator. Race/ethnicity was categorized as household/parental characteristics because the HSIS reports defined subgroups using information on biological mother's or caregiver's race/ethnicity, rather than children's race/ethnicity. Thirty-eight percent used parental education level and marital status, 31 percent used household income, and 23 percent used parental age and parental depressive symptoms. Eight studies (29%) explored child care type as a moderator. Feller et al. (2016), Kline and Walters (2016), and Zhai, Brroks-Gunn, & Waldfogel (2014) estimated the Head Start effect by counterfactual, or alternative care types, such as center-based care compliers (i.e., those who would have attended Head Start if it were offered and otherwise, would have attended another child care center) and home-based care compliers (i.e., those who would have attended Head Start if it were offered but otherwise, would have been cared at home). Other studies used child care types recorded at baseline, such as non-parental care.

Four studies (18%) explored TEH by Head Start center characteristics. Miller (2017) and Walters (2015) considered center characteristics separately, such as teacher's education level, student/staff ratio, and the curriculum, while Lee (2019) used a composite measure for the center quality.

Two studies (9%) used urbanicity as a moderator, a neighborhood characteristic. To assess differential Head Start effects by urbanicity, Sabol and Chase-Lansdale (2015) considered whether children lived in urban versus nonurban areas, while McCoy et al. (2016) stratified by whether children's Head Start centers were located in urban, mixed, or nonurban areas. No other neighborhood moderators were explored.

#### 3.1.3 Targeted cohort and assessment year

The studies analyzed the HSIS cohorts in four distinctive ways: 1) a 3-year-old cohort only (29%; n = 8), 2) a 4-year-old cohort only (21%; n = 6), 3) two cohorts combined (82%; n = 23) by time since the start of Head Start or 4) by academic year (Table A4). For example, when the cohorts were combined by time since Head Start, Year 1 is age 3 for the 3-year-old cohort and age 4 for the 4-year-old cohort. Alternatively, the two cohorts were combined by academic year, meaning that they were matched by their age. For analyses separate for each 3- and 4-year-old cohort, Year 1 was studied most frequently (88% or n = 7 for the 3-year-old cohort; 100% or n = 6 for the 4-year-old cohort), while third grade year was never studied. The combined cohort was studied most frequently at Year 1 (68%; n = 15) and Year 3 (39%; n = 9).

#### 3.1.4 Analytic methods: parameter of interest, causal estimand, and attrition

The studies did not vary much in parameters of interest when assessing TEH. All studies except two assessed TEH by comparing ATEs across subgroups (Table 1; 93%; n = 26). The ATE for each subgroup was estimated by either restricting the analysis to that subgroup (i.e., subgroup analysis) or including a model parameter which assessed interaction between Head Start and the moderator (i.e., interaction analysis). Alternative methods included estimation of ATE in subgroups defined by post-randomization counterfactual care types in a principal stratification framework (Feller et al., 2016; Zhai et al., 2014), and structural equation modeling methods, such as path analysis (Lipscomb et al., 2013), latent class analysis (Cooper & Lanza, 2014), and multiple group analysis (Ansari et al., 2016).

Few studies considered distributional effects, in a form of QTE or variance. Two studies (7%), Bitler et al. (2014) and Feller and others (2016), estimated QTE using a quantile regression. QTE is the difference in quantiles of outcome distribution between a treatment group and a control group, which captures how each quantile of distribution is affected, rather than just the mean (Koenker & Bassett, 1978). If the treatment effect were constant across all individuals, the QTE estimates would be equal for all quantiles. On the other hand, varying QTE estimates by quantiles suggests the presence of TEH. Two other studies (7%) utilized variance estimates to assess TEH. Bloom and Weiland (2015) and Walters (2015) used two under-utilized features of a multilevel model in the assessment of TEH. First, they estimated variation in center-specific Head Start effect (i.e., between-center variation in ATE). In addition to accounting for clustering due to a hierarchical nature of data (e.g., children (level 1) nested in centers (level 2) which are nested in programs agencies (level 3) in the HSIS data), multilevel models can also estimate the amount of variability in treatment effects (i.e., TEH) in each higher-level unit (e.g., centers) in the form known as a random slopes model. In the HSIS data, the ATE is modeled in each center, and the variation in these center-specific ATEs quantifies the amount of TEH. Second, Bloom and Weiland (2015) compared the effect of Head Start on variance of outcome (i.e., within-center/between-child variation in outcome) in the treatment and control groups. By relaxing the assumption of constant variance in a standard ordinary least squares regression, multilevel models can model variance as a function of covariates (Goldstein, 2011). In the HSIS data, variance can be estimated separately in the treatment and control groups. Substantial differences in these variance estimates after Head Start indicate that Head Start had heterogeneous effects on children. Ding et al. (2016, 2019) developed statistical methods for RCT settings to assess the presence of meaningful TEH by considering individual treatment effects. They tested, without directly estimating, whether there is variation in treatment effects across individuals and quantified the proportions of a systematic component and an idiosyncratic component of the total TEH. The systematic components represent a part of the treatment effect variation that is explained by all observed covariates in the HSIS data, and the idiosyncratic component represents the unexplained remainder.

Most studies used the weights provided by the HSIS reports to adjust for attrition over the follow-up periods, while some used imputation (Table A1). A few studies did not adjust for the attrition, but these results should be taken with care because about 10–30 percent of children were lost depending on the follow-ups and outcomes (Puma et al., 2010). In terms of the causal estimand of interest, most studies took intent-to-treat (ITT) approach in which they used a random assignment to Head Start (i.e., treatment assignment), not the enrollment to Head Start (i.e., treatment compliance), as the treatment variable. The advantage of the ITT approach is that it prevents confounding between the treatment and outcomes by preserving the randomized nature of the treatment in the sample. However, a substantial amount of crossover between the treatment and control groups occurred; in 3- and 4-year-old cohorts, 14.9 percent and 20.2 percent of the Head Start children did not actually attend Head Start, and 17.3 percent and 13.9 percent of the control children attended Head Start (Puma et al., 2010). To amend this problem, some studies took treatment-on-the-treated (TOT) approach and applied the instrumental variable estimation to adjust for confounding. A small number of studies offered TOT effect estimates without any adjustment (“endogenous TOT”), and these studies should be interpreted with caution. Endogenous TOT estimates would be biased if children who did not comply with the random treatment assignment are systematically different from those who did comply with the assignment.

### 3.2 Study findings

Moderators that were examined in at least 3 studies and thus included in qualitative summaries of treatment effect finding were baseline cognitive skills, language, special needs status, gender, race/ethnicity, household income, parental educational attainment, marital status, parental age, parental depressive symptoms, non-parental care at baseline, and counterfactual care type. Study findings on these moderators were organized into subgroups with greater beneficial treatment effects that were relatively consistent across multiple outcomes (Table 2) and subgroups with treatment effects that were inconsistent across outcomes (Table 3). Note that Table 2, Table 3 are not meant to be comprehensive and should be treated as visual aids. To readily visualize the patterns of treatment effects for each moderator by each assessment year, studies that analyzed outcomes by a combination of two or more moderators (e.g., effect moderation by gender among children who had non-parental care at baseline) were excluded. Findings by Gelber and Isen (2013) at “After period” were also excluded because they combined the outcomes across three years (i.e., age 4, kindergarten, and 1st grade). Nonetheless, these exclusions did not distort the representation of the general trends in study findings, and all relevant studies without an exception are summarized in the main text below.

**Table 2:** Subgroups with beneficial treatment effects that are consistent across multiple outcomes.

	Year since Head Start (combined cohort – 1st, 2nd, and 3rd year)			Academic Year (combined cohort – age 4, kindergarten, 1st and 3rd grade; 3-year-old cohort – age 3, age 4, kindergarten, 1st and 3rd grade; 4-year-old cohort – age 4, kindergarten, 1st and 3rd grade)				
Moderator	Year 1	Year 2	Year 3	Age 3	Age 4	Kindergarten	1st Grade	3rd Grade
Low baseline cognitive measures	Walter (2015): **↑**,**↑** Bloom and Weiland (2015): **+↑**,**+≡**,**≈≡**,**+↑,≈≡**,**≈≡** Miller et al. (2016): **≡,≡,≡,≡,≡,≡**	n/a	Bloom and Weiland (2015): **≈≡**,**≈↑**	Bitler et al. (2014): **+**	n/a	Miller et al. (2016): **≡,≡,≡,≡,≡,≡,≡,≡,≡**	n/a	n/a
Spanish-speaking dual language learners	Bloom and Weiland (2015): **+↑**,**+≡**,**≈≡**,**+↑,≈≡**,**≈≡** Miller (2017): **+↑**,**≈≡**,**+≡**	n/a	n/a	Bitler et al. (2014): **+**	n/a	n/a	n/a	n/a
Girls	Bloom and Weiland (2015): **+≡,+≡,≈≡**,**+↑,+↑** Gelber and Isen (2013): **+**	n/a	n/a	n/a	n/a	n/a	n/a	n/a
Hispanic	Bloom and Weiland (2015): **+↑**,**+≡**,**≈≡**,**+↑**,**≈≡**,**≈≡** Gelber and Isen (2013): **+**	n/a	n/a	Bitler et al. (2014): **+** Sabol and Chase-Lansdale (2015): **≈≡**	Sabol and Chase-Lansdale (2015): **≈≡**,**≈↓**	Sabol and Chase-Lansdale (2015): **≈↓**,**≈≡**	Sabol and Chase-Lansdale (2015): **≈↓**,**≈≡**	n/a
Blacks	Bloom and Weiland (2015): **≈↓**,**+≡**,**≈≡**,**≈↓**,**≈≡**,**≈≡** Gelber and Isen (2013): **+**	n/a	n/a	Bitler et al. (2014): **≈** Sabol and Chase-Lansdale (2015): **≈≡**	Sabol and Chase-Lansdale (2015): **≈≡**,**+↑**	Sabol and Chase-Lansdale (2015): **+↑**,**≈≡**	Sabol and Chase-Lansdale (2015): **≈↑**,**≈≡**	
Low education level	Walter (2015): **↑**,**↑**	n/a	n/a	Long (2015): **≈,+,≈,+,≈,≈,≈,≈** Sabol and Chase-Lansdale (2015): **≈,≈,+,≈**	Long (2015): **≈,≈,≈,≈,≈,≈,≈,≈** Sabol and Chase-Lansdale (2015): **≈,≈,≈,≈,+,≈**	Sabol and Chase-Lansdale (2015): **≈,≈,≈,≈,+,≈**	Sabol and Chase-Lansdale (2015): **≈,≈,≈,≈,+**	n/a
Home-based care compliers	Kline and Walters (2016): **+**	n/a	n/a	Feller et al. (2016): **+** Zhai et al. (2014): **+**,**+**,**+**,**≈**,**≈**,**+**	Feller et al. (2016): **+**,**+** Zhai et al. (2014): **≈**,**+**,**+**,**≈**,**+**,**+**,**+,+**,**+,≈,≈**,**+**	Feller et al. (2016): **≈**,**≈** Zhai et al. (2014): **≈,≈,≈,≈**,**+**,**+**,**+**,**≈**,**+,≈,≈,≈**	Feller et al. (2016): **≈**,**+** Zhai et al. (2014): **≈,≈**,**+**,**≈,≈**,**+**,**+**,**+**,**+,≈,≈,≈**	n/a
Non-parental care at baseline	Lipscomb et al. (2013): **+**,**+**,**≈** Pratt et al. (2015): **+,≈,≈,≈,+**	n/a	Lee and Lee (2016): **≈,≈,≈,≈,≈,+** Lee (2016): **≈,≈** Lee (2020): **+,≈,≈,≈**	n/a	n/a	n/a	n/a	Lee (2020): **+**,**+,≈,≈**

The study finding for each outcome is represented as a pair of symbols or a single symbol based on the results of subgroup and/or interaction analysis results. Analyses of combinations of two or more moderators (e.g., assessing effect moderation by gender within children who had non-parental care at baseline) were excluded to summarize the findings by each moderator. The studies are organized by the academic year of the targeted cohort, except for the studies that analyzed cohorts combined by year since Head Start assignment. Year 1 was at the end of age 3 for the 3-year-old cohort and age 4 for the 4-year-old cohort. Year 2 was at the end of age 4 for the 3-year-old cohort and kindergarten for the 4-year-old cohort. Year 3 was at the end of kindergarten for the 3-year-old cohort and 1st grade for the 4-year-old cohort.

Subgroup analysis: **+** (treatment effect >0, p < 0.05), **≈** (p > 0.05); Interaction analysis: **↑** (treatment effect > other subgroups, p < 0.05), **≡** (p > 0.05), **↓** (treatment effect < other subgroups, p < 0.05); Example: Hispanic (**+≡**): at the 0.05 level of significance, the treatment effect for Hispanic children was beneficial but not different from treatment effects for other subgroups, such as White and Black children.

**Table 3:** Subgroups with treatment effects that are inconsistent across multiple outcomes.

	Year since Head Start (combined cohort – 1st, 2nd, and 3rd year)			Academic Year (combined cohort – age 4, kindergarten, 1st and 3rd grade; 3-year-old cohort – age 3, age 4, kindergarten, 1st and 3rd grade; 4-year-old cohort – age 4, kindergarten, 1st and 3rd grade)				
Moderator	Year 1	Year 2	Year 3	Age 3	Age 4	Kindergarten	1st Grade	3rd Grade
Special needs	Bloom and Weiland (2015): **+≡**,**≈≡**,**≈≡**,**≈≡**,**≈≡**,**≈≡** Shapiro and Weiland (2019): **+≡, +≡,≈≡**,**≈≡**,**≈≡**,**≈≡**,**≈≡**,**≈≡**,**≈≡**,**≈≡**,**≈↑**,**≈≡**	n/a	Lee and Rispoli (2016): **≈,≈,≈,≈,≈** Lee et al. (2016): **≈,≈,≈,≈,≈**	n/a	n/a	n/a	Shapiro and Weiland (2019): **+≡, ≈↑,≈≡**,**≈≡**,**≈≡**,**≈≡**,**≈≡**,**≈≡**,**≈≡**,**≈≡**,**≈≡**,**≈≡**	Shapiro and Weiland (2019): **≈≡, ≈≡,≈≡**,**≈≡**,**≈≡**,**≈≡**,**≈≡**,**≈≡**,**≈≡**,**≈≡**,**≈↑**,**≈≡**
Low household income	Walter (2015): **↑**,**↑**	n/a	n/a	Sabol and Chase-Lansdale (2015): **≈≡**	Sabol and Chase-Lansdale (2015): **≈≡**,**≈≡**	Miller et al. (2016): **≡,≡,≡** Sabol and Chase-Lansdale (2015): **≈≡**,**≈≡**	Sabol and Chase-Lansdale (2015): **≈≡**,**≈≡**	n/a
Single parent	Gelber and Isen (2013): **+**	n/a	n/a	Sabol and Chase-Lansdale (2015): **≈≡** Long (2015): **≈,≈,≈,+,≈,≈,≈,≈**	Sabol and Chase-Lansdale (2015): **≈≡**,**≈≡** Long (2015): **≈,≈,≈,≈,≈,≈,≈,≈**	Sabol and Chase-Lansdale (2015): **≈≡**,**≈≡**	Sabol and Chase-Lansdale (2015): **≈≡**,**≈≡**	n/a
Younger caregivers	n/a	n/a	n/a	Sabol and Chase-Lansdale (2015): **≈≡**	Sabol and Chase-Lansdale (2015): **≈≡**,**≈≡**	Sabol and Chase-Lansdale (2015): **+≡**,**≈≡**	Sabol and Chase-Lansdale (2015): **+≡**,**≈≡**	n/a
Caregivers with depressive symptoms	Miller et al. (2016): **≡,≡**	n/a	n/a	Ansari et al. (2016): **+↑**	Ansari et al. (2016): **≈≡**	n/a	n/a	n/a

The study finding for each outcome is represented as a pair of symbols or a single symbol based on the results of subgroup and/or interaction analysis results. Analyses of combinations of two or more moderators (e.g., assessing effect moderation by gender within children who had non-parental care at baseline) were excluded to summarize the findings by each moderator. The studies are organized by the academic year of the targeted cohort, except for the studies that analyzed cohorts combined by year since Head Start assignment. Year 1 was at the end of age 3 for the 3-year-old cohort and age 4 for the 4-year-old cohort. Year 2 was at the end of age 4 for the 3-year-old cohort and kindergarten for the 4-year-old cohort. Year 3 was at the end of kindergarten for the 3-year-old cohort and 1st grade for the 4-year-old cohort.

Subgroup analysis: **+** (treatment effect >0, p < 0.05), **≈** (p > 0.05); Interaction analysis: **↑** (treatment effect > other subgroups, p < 0.05), **≡** (p > 0.05), **↓** (treatment effect < other subgroups, p < 0.05); Example: Hispanic (**+≡**): at the 0.05 level of significance, the treatment effect for Hispanic children was beneficial but not different from treatment effects for other subgroups, such as White and Black children.

#### 3.2.1 Subgroups that benefitted consistently across multiple outcomes

Among subgroups defined by child characteristics, treatment effects were consistently larger and beneficial across multiple outcomes for children with lower cognitive skills at baseline, Spanish-speaking dual language learners, and girls (Table 2). Children with lower cognitive skills at baseline appeared to have benefitted more from Head Start for a range of cognitive outcomes, especially for PPVT, compared to those with higher cognitive skills at baseline (Bitler et al., 2014; Bloom & Weiland, 2015; Feller et al., 2016). When composite cognitive measures were used as outcomes, the effects were attenuated but still larger for those with lower baseline cognitive skills (Miller et al., 2016; Walters, 2015). Unexpectedly, among children with non-parental care at baseline, the beneficial effect on the frequency of parental book reading for the child was larger for children with higher baseline cognitive skills (Lee & Lee, 2016). For Spanish-speaking dual language learners, multiple studies found larger beneficial effects of Head Start on various cognitive outcomes (Bitler et al., 2014; Bloom & Weiland, 2015; Feller et al., 2016; Miller, 2017). Cooper and Lanza (2014) also found that latent class subgroups with dual language learners had larger beneficial effects on both cognitive and social-emotional outcomes. For both girls and boys, Head Start had beneficial effects on their cognitive outcomes (Bloom & Weiland, 2015) and their parents’ parenting activities (Gelber & Isen, 2013). Girls improved their externalizing behaviors and self-regulation measures, while boys had no benefits (Bloom & Weiland, 2015). Among children with non-parental care at baseline, girls improved more on reading and math scores than boys, while boys had math scores even lower than their counterparts who did not get assigned to Head Start (i.e., the control group) (Lee, 2016).

Among subgroups defined by household/parental characteristics, Hispanics, Blacks, and children with low parental education level had larger beneficial treatment effects that were relatively consistent across outcomes. Head Start was more effective for children with Hispanic parents compared to White and Black parents on several cognitive and social-emotional outcomes (Bloom & Weiland, 2015). Among children with special needs, children with Hispanic and Black parents had larger beneficial Head Start effects than children with White parents on social-emotional outcomes (Lee et al., 2016). Head Start also increased Hispanic and Black parents’ parenting activities and involvement, and this effect lasted even after the year of Head Start for Hispanic parents (Gelber & Isen, 2013). Additionally, Black parents were able to advance their education when their children were assigned to Head Start (Sabol & Chase-Lansdale, 2015). Children who had parents with education level of high school or less (i.e., low education level) gained more from Head Start on cognitive and social-emotional outcomes (Cooper & Lanza, 2014; Walters, 2015). At the end of Head Start program (i.e., Year 1), these parents were more likely to be employed in a full-time job and have enrolled in educational courses (Long, 2015). Parents with some college experience but without degrees were able to advance their education and earn degrees if their children were assigned to Head Start (Sabol & Chase-Lansdale, 2015).

Home-based care compliers and those with non-parental care at baseline were child care types in which children benefitted more from Head Start consistently. For children with non-parental care at baseline, the beneficial Head Start effect was statistically significant at Year 1 when assessed with a composite cognitive outcome (Lipscomb et al., 2013), although the effect was faded at Year 3 when assessed separately on math and reading skills (Lee, 2016). Also at Year 1, Head Start increased non-parental care children's health services and parents' preschool-based involvement, and decreased the frequency of the parents' physical discipline on children (Pratt et al., 2015). These parents did not read more frequently for their children at Year 3 (Lee & Lee, 2016). However, all long-term findings for children with non-parental care at baseline were from endogenous TOT effect estimates and did not adjust for attrition and selection, which raise questions for its validity (Lee, 2016, 2020; Lee & Lee, 2016). In addition, multiple studies confirmed that home-based care compliers benefitted much more from Head Start on cognitive and social-emotional outcomes, with some effects lasting until first grade (Feller et al., 2016; Kline & Walters, 2016; Zhai et al., 2014). Moreover, these studies also collectively found that the Head Start effects were much smaller and statistically insignificant for center-based care compliers for cognitive and social-emotional outcomes.

#### 3.2.2 Subgroups with mixed results on different outcomes

There were no clear patterns in treatment effect findings by children with special needs status, low household income, single parents, younger caregivers, or caregivers with depressive symptoms (Table 3). Bloom and Weiland (2015) and Shapiro and Weiland (2019) found benefits for special needs children on PPVT, other studies showed no differential benefits for special needs children on a range of other cognitive outcomes (Lee & Rispoli, 2016) and social-emotional outcomes (Lee et al., 2016). However, the discrepancy may have occurred because the latter studies may have been biased as they only offered endogenous TOT effect estimates and did not adjust for attrition and selection. In contrast to non-special needs children, those with special needs did not benefit on math skills, Letter-Word Identification, and externalizing behaviors (Shapiro & Weiland, 2019). Among non-parental care children at baseline, special needs children experienced a marginally higher frequency of parental book reading for the child (Lee & Lee, 2016). Special needs children with higher household income had larger beneficial effects on social-emotional outcomes relative to those with low household income (Lee et al., 2016). Walters (2015) found a slightly larger effect on cognitive outcomes for children with lower household income, but household income did not matter for increasing parents' educational advancement (Sabol & Chase-Lansdale, 2015). Compared to children with married parents, children with single parents didn't have differential benefits on cognitive outcomes, but had larger beneficial effects for social-emotional outcomes (Cooper & Lanza, 2014). The single parents didn't have differential benefits on parenting activities (Gelber & Isen, 2013), and they neither advanced their education (Sabol & Chase-Lansdale, 2015) nor secured full-time employment (Long, 2015), while the married parents did. They did enroll in educational courses more than married parents (Long, 2015). For children with parents who were younger (Lee, 2016) or had depressive symptoms (Miller et al., 2016), the effects on cognitive outcomes were not statistically significant. Children with parents who had depressive symptoms at baseline were negatively affected by Head Start on social-emotional outcomes (Cooper & Lanza, 2014) but these parents alleviated their symptoms after one year of Head Start (Ansari et al., 2016). Younger parents were able to advance their education due to their children's Head Start assignment (Sabol & Chase-Lansdale, 2015).

#### 3.2.3 Distributional effects

Head Start had a larger QTE on the lower part of cognitive outcome distribution, resulting in a decreased overall variation in cognitive abilities among children (Bitler et al., 2014; Feller et al., 2016). This suggests that there may have been larger benefits for those who had lower cognitive ability at baseline. Such pattern persisted when the sample were stratified by baseline cognitive skills, age, primary language, parent's race/ethnicity, and counterfactual care types. Treatment effects also varied substantially across the Head Start centers (Bloom & Weiland, 2015; Walters, 2015). Head Start also reduced variance, or the overall dispersion, in cognitive outcomes after the end of Head Start (Bloom & Weiland, 2015).

#### 3.2.4 Explained and unexplained proportions of treatment effect heterogeneity

Ding and others (2016, 2019) found that there was indeed substantial variation in treatment effects. They reported that first, observed covariates in the HSIS accounted for a large amount of treatment effect variation, meaning that these covariates may be the major source of TEH, and second, a large amount of treatment effect variation remained even after accounting for these covariates, suggesting that other important but unobserved sources of TEH exist.

The estimated proportions of treatment effect variation explained by the covariates (i.e., R^2^-like estimate) depends on the assumptions about how much Head Start affected the individuals’ ranks in the outcome distribution. The authors found that if the correlation between ranks of the two potential outcomes (i.e., treatment vs. control) equals one (i.e., the ranks are preserved), the R^2^-like estimate was 0.76, and if the correlation is zero, the R^2^-like estimate was 0.03. This rank-preserving feature of an intervention is generally assumed to be strong (i.e., closer to 1 than 0), and therefore, a substantial amount of the treatment effect variation appeared to be explained by the observed covariates. In particular, having a mother who is a recent immigrant and Spanish-speaking dual language learner status were two characteristics that explained the variation the most. In addition, the authors found that the non-compliance did not explain much of the variation with the R^2^-like estimates ranging from 0.01 to 0.16, indicating that there was a large amount of TEH even after accounting for the observed covariates and non-compliance.

#### 3.2.5 Overall trends of study findings

When all studies were combined, 24 percent (264/1118) of the hypotheses tested were statistically significant, indicating a substantial amount of TEH (Table 4). Among the outcome categories, cognitive outcomes had the highest proportions (34%; 146/426) when health outcomes are excluded (80%; 4/5) for which only two studies were available. Social-emotional outcomes (18%; 54/308) and parental outcomes (16%; 58/357) had similar proportions of statistically significant findings. Among the moderator categories, child characteristics had the highest proportions of statistically significant findings (30%; 95/318), while there were similar proportions across household/parental characteristics (19%; 89/467), neighborhood characteristics (26%; 8/31), and child care type (26%; 56/218).

**Table 4:** Proportions of statistically significant findings by outcome and moderator categories.

Outcome Category	Cognitive	Social-emotional	Health	Parental	Total
Moderator Category					
**Child characteristics**	64/187 (34%)	25/121 (21%)	n/a	6/10 (60%)	95/318 (30%)
**Household/parental characteristics**	37/84 (44%)	8/73 (11%)	n/a	44/310 (14%)	89/467 (19%)
**Neighborhood characteristics**	4/10 (40%)	n/a	n/a	4/21 (19%)	8/31 (26%)
**Center characteristics**	2/11 (18%)	1/11 (9%)	n/a	n/a	3/22 (14%)
**Child care type**	29/108 (27%)	20/98 (20%)	4/5 (80%)	3/7 (43%)	56/218 (26%)
**Combination of moderator categories**^a^	10/26 (38%)	4/29 (14%)	n/a	1/9 (11%)	15/64 (23%)
**Total**	146/426 (34%)	58/332 (17%)	4/5 (80%)	58/357 (16%)	264/1118 (24%)

a Some studies combined two or more moderators (e.g., dual language learner whose mother has severe depressive symptoms).

For 3-year-old cohort, the highest proportion of statistically significant findings was found at the end of age 3 (27%; 29/108) (Table 5). For 4-year-old cohort, the proportions were comparable at the end of age 4, kindergarten, and first grade. For the combined cohort, the highest proportions were found in Year 1 (41%; 116/283) when analyzed by time since Head Start, and in first grade (21%; 5/24) when analyzed by academic year. When all studies were aggregated by time since Head Start, Year 1 (30%; 170/560) had the highest statistically significant proportions. In contrast, when all studies were aggregated by academic year, the proportions were comparable across age 4 (17%; 56/323), kindergarten (13%; 36/277), first grade (15%; 40/271), and third grade (17%; 9/54).

**Table 5:** Proportions of statistically significant findings by targeted cohort and assessment year.

**3-year-old cohort**							
**Age 3**	**Age 4**	**Kindergarten**	**1st grade**	**3rd grade**			
41/147 (28%)	41/191 (21%)	27/191 (14%)	27/191 (14%)	n/a			

**4-year-old cohort**							
**Age 4**	**Kindergarten**	**1st grade**	**3rd grade**				
15/132 (11%)	5/56 (9%)	8/56 (14%)	n/a				

**Combined cohort by 1) time since Head Start or 2) academic year**							
**1) Time since Head Start**				**2) Academic year**^a^			
**Year 1**	**Year 2**	**Year 3**		**Age 4**	**Kindergarten**	**1st grade**	**3rd grade**
170/560 (30%)	52/280 (19%)	59/412 (14%)		56/323 (17%)	36/277 (13%)	40/271 (15%)	9/54 (17%)

**All studies combined by 1) time since Head Start or 2) academic year**							
**1) Time since Head Start**				**2) Academic year**			
**Year 1**	**Year 2**	**Year 3**		**Age 4**	**Kindergarten**	**1st grade**	**3rd grade**
170/560 (30%)	52/280 (19%)	59/412 (14%)		56/323 (17%)	36/277 (13%)	40/271 (15%)	9/54 (17%)

a The analysis that combined age 4 to first grade was not included in this table (Gelber & Isen, 2013).

## 4 Discussion

This review provides five salient findings from a comprehensive synthesis of studies on heterogeneous effects of Head Start using the HSIS data. First, we identified multiple subgroups of children and their parents who experienced greater benefits from Head Start across multiple outcomes, while for other subgroups, the results were mixed. Second, most studies focused on conventional subgroup or interaction analyses, while distributional effects remain largely underexplored. Third, of all the outcomes measured in the HSIS, cognitive and social-emotional outcomes were primarily assessed for TEH whereas evidence for health and parental outcomes are largely missing. Fourth, a large proportion of the included studies used baseline child or household/parent characteristics as moderators. We also found that higher proportions of study findings were statistically significant for cognitive outcomes or by child characteristics. Lastly, most studies on TEH were restricted to assessment of short-term effects, although analyses of short-term and long-term TEH had comparable proportions of statistically significant findings.

Based on the findings on ATEs, subgroups that consistently benefitted from Head Start across multiple developmental and parental outcomes were children who had lower cognitive skills at baseline, limited English ability, less educated parents, or non-parental care at baseline, children who were female, Hispanic, or Black, or those who would have been cared at home if not assigned to Head Start. Most of these subgroups are at a greater disadvantage than others, indicating that Head Start had compensatory effects, or greater impacts for those who are in need the most (Sameroff, A. & Chandler, M., 1975). Head Start may have been particularly effective for children with least resources because they received services that were far better than their alternatives. For example, children who had lower cognitive skills or limited English ability at baseline may have lacked necessary resources at home to develop English language skills and cognitive ability, which Head Start provided. The minimal effects for center-based care compliers (i.e., those who would have received other center-based care, if not assigned to Head Start) also supports the compensatory hypothesis; they would have received necessary care regardless of their assignment to Head Start. The findings on distributional effects and variance strengthens these ATE findings. Larger effects at lower quantiles of cognitive outcome distributions and reduction in total between-child variability of cognitive outcomes both suggest that Head Start benefitted those who were more disadvantaged and alleviated the inequality in cognitive performance by pulling forward those that were left behind. These distributional effect findings are aligned with the findings of previous research on child development interventions (Duncan & Sojourner, 2013; Magnuson et al., 2004; McCartney et al., 2007). Differential noncompliance and counterfactual care types did not explain away the larger favorable impacts for children who had lower cognitive skills or who were Spanish-speaking dual language learners (Bitler et al., 2014; Bloom & Weiland, 2015).

Given that Head Start already targets low-income families, the evident compensatory effects within this already high-risk group suggests that this phenomenon lies in a continuum and extends beyond the simple categorization of a social disadvantage by income. Moreover, the amount of TEH found across the studies reveals the complex nature of childhood development and early developmental interventions. Recognizing that individual experiences occur at the intersection of multiple social disadvantages (Collins & Bilge, 2016; Crenshaw, 1989), Cooper and Lanza (2014) analyzed the Head Start effects in subgroups defined by multiple individual and family characteristics, and confirmed a more nuanced case of TEH. For example, they found that among children who spoke Spanish at home and had less educated parents, those with married parents benefitted in cognitive outcomes more than those with single parents. Mixed findings for children with special needs, low household income, single parents, younger parents, or parents who have depressive symptoms also demonstrate the limitations of single-moderator analyses for ATEs.

Nonetheless, we found that the number of ATE analyses across subgroups dominated the current state of evaluating TEH in the HSIS data. Reliance on the evidence from subgroup ATEs for designing and evaluating programs and policies is often insufficient and can be misleading (Subramanian et al., 2018). Bitler et al. (2017) has showed that conventional subgroup analyses can fail to capture TEH found in distributional effect analyses, suggesting that variation in subgroup-specific ATEs alone are inadequate to characterize the overall phenomenon of TEH. Furthermore, Ding and others (2016, 2019) found substantial TEH in Head Start beyond what the observed covariates and noncompliance can explain, meaning that different approaches need to be taken to better understand for whom Head Start is effective.

Distributional effects are rarely examined, either by estimating QTEs or comparing post-treatment variances of outcomes in the treatment and control groups. Studies with such methods are especially useful in RCT settings like the HSIS, where not only the means, but also the dispersion of outcome distribution at baseline should be balanced across the treatment and control groups. Consequently, after the intervention, meaningful amount of heterogeneity in QTEs or difference in the variances of outcomes between comparison groups would indicate that there are substantively important TEH to uncover. If the outcome distribution had been narrowed, the intervention would be considered capable of reducing inequality in that outcome. On the other hand, if the outcome distribution had been widened, the intervention may be unexpectedly exacerbating the existing inequality due to its ineffectiveness for certain subgroups (Subramanian et al., 2018).

A disproportionate number of studies focused on a small number of cognitive and social-emotional outcomes. Health and parental outcomes have not received much attention in terms of the number of studies, although the number of hypotheses tested for parental outcomes is comparable to social-emotional outcomes. Healthcare, nutritional, and parental services were important parts of Head Start, but their effectiveness remains unclear. Even the two studies on health outcomes only reported the effect on health services, rather than child's health status, possibly due to minimal data collected on health outcomes. Inactive research on the health impacts of Head Start using the RCT data is unfortunate since pre-HSIS findings on the health impact of Head Start were also sparse (Zigler et al., 1994). Besides, the established evidence on the impact of physical development and parenting in early childhood on children's life trajectories and well-being warrants more research on health and parental outcomes (Bowers et al., 2012). A potential explanation for the currently skewed investigations focused on a small number of outcomes may be due to the more pronounced effects in these outcomes found in the HSIS reports. However, this is problematic because large heterogeneity in treatment effects appeared to be masked in null ATEs presented in the HSIS reports. For example, Bloom and Weiland (2015) found a substantial cross-center variation in the effects even in outcomes with null ATEs. Another potential reason may be that many outcomes were non-standardized, had limited psychometric properties, and had scoring issues reported by the HSIS reports. For example, Color Identification, Counting Bears, and Letter Naming do not have published reliability measures.

Short-term effects have been examined at a much higher frequency, even though previous studies on Head Start and other child development interventions found long-term effects (Deming, 2009; Garces et al., 2002; Havnes & Mogstad, 2011; Ludwig & Miller, 2007; Steven Barnett, 1995). Indeed, when studies were combined by academic year, we showed that the proportions of statistically significant findings were comparable in the early and later follow-ups. With only three studies assessing the effects at third grade, the outcomes which were unique to the third grade follow-up, such as teacher-reported children's strengths and difficulties measures and children's self-reported academic and social skills, have not been examined. In addition, although the HSIS follow-up ended at the children's third grade year, an extended follow-up for young adulthood outcomes would be worthwhile for understanding the long-term Head Start impacts given that previous observational studies suggest that Head Start can improve a range of adulthood outcomes, including high school graduation rate, college attendance, income, career-related productiveness, health status, crime rate, and teen pregnancy (Deming, 2009; Garces et al., 2002). These long-term benefits were found despite the quickly faded-out short-term effects on developmental outcomes. As such, the rapid decline in short-term ATEs in the HSIS cohorts may not be such disappointing results; they could well be meaningful proxies for long-term adulthood benefits (Deming, 2009; Ludwig & Phillips, 2008).

The mechanisms through which early child development interventions improve long-term outcomes are currently unclear. The long-term effects described above were larger for more disadvantaged subgroups (Deming, 2009; Garces et al., 2002). If the same phenomenon applies to the HSIS cohorts, we may find that the high-risk group with larger short-term gains also experience larger long-term gains. Alternatively, as some suggested that improvement in social-emotional outcomes (Gibbs et al., 2012) or parental outcomes (Gelber & Isen, 2013) mediate the impact of early childhood intervention on long-term outcomes, we may find subgroups with larger short-term gains in these specific outcomes to experience larger long-term gains. However, whichever the mechanism, we do not expect these patterns to be homogeneous across subgroups. Future research should test the pattern of compensatory effects in long-term and uncover the heterogeneity in mediating mechanisms of long-term Head Start effects.

Most studies explored how Head Start effects varied by baseline child and household/parent characteristics. Alternatively, other studies showed that the counterfactual care type is a significant moderator. Counterfactual care types can be determined by not only individual participant's characteristics, but also local child care alternatives. For example, child care licensing regulations vary greatly across states, and more rigorous regulations have been associated with improved access and quality of Head Start and other child care programs (Connors & Friedman-Krauss, 2017). This also necessitates more attention in the effort to discover contextual factors that can be modified at neighborhood- and state-level to assist the effort of Head Start in ensuring children's healthy development. The quantitative summary showed that the statistically significant proportion of the study findings was relatively high for neighborhood characteristics, compared to other moderators. Moreover, McCoy and others (2016) found meaningful heterogeneity by urbanicity, even after taking account of individual compositional characteristics. In addition to the contextual factors, the Head Start center quality may be a driver of large variation in the effect of Head Start observed across centers (Bloom & Weiland, 2015; Walters, 2015). While an HSIS report on center quality (Peck & Bell, 2014) did not find convincing evidence that composite measure of center quality matters for program effects, studies in this review found some center quality measures, such as full-day service, frequent home visits, and Spanish instructions for Spanish-speaking children, to be important moderators. Walters (2015) also found that center quality measures that are considered effective, such as highly educated teachers or low student:staff ratio, did not magnify the effectiveness of Head Start. More studies on center quality are needed to provide clearer guidance on how to construct each Head Start center to maximize its impact, especially given the highly varying qualities across the Head Start center (Puma et al., 2010).

This review encountered several limitations. First, we aggregated each characteristic into wider categories (e.g., outcomes into outcome categories, moderators into moderator categories). While it is helpful to evaluate the overall trend of statistically significant findings, such approach may have masked important patterns in study findings. Second, we relied on published studies, acknowledging that publication bias may be present, especially given the high number of outcomes present in the HSIS data. Third, some studies restricted the sample to subgroups with small sample sizes. Relying on statistical significance to summarize the overall trend may be tainted by low statistical power, which may have concealed meaningful heterogeneity. With these three limitations in mind, the quantitative summaries of the study findings should be interpreted with caution; it is a descriptive statistic, rather than formally tested evidence of TEH. Fourth, for multiple subgroups, we found that the effects were inconsistent across outcomes. While it is reasonable for a subgroup to benefit on one outcome while experiencing a null effect on the other outcome, we do not infer any trend from heterogeneous effects across outcomes for a given subgroup and consider this beyond the scope of the review. Lastly, the evidence for TEH synthesized in this review is from the Head Start program in 2002–2003, and the findings may not directly generalize to the Head Start implementation today. Nonetheless, the comprehensive review from the past findings is essential, and the lessons learned are applicable to future research for Head Start or other child developmental interventions.

Collectively, we found substantial variation in the effects of Head Start by Head Start centers, by subgroups, and by individuals. We identified several subgroups with larger gains consistently across multiple outcomes. The findings on ATEs and distributional effects suggest the compensatory effects of Head Start; Head Start benefitted high-risk subgroups more and reduced the overall inequality in outcomes. However, the mixed findings for some high-risk subgroups and the findings on latent subgroups defined by multiple dimensions of social disadvantages represent a more nuanced reality of the Head Start effects and underscore corresponding analytical challenges. In a similar vein, the current reliance on single-moderator analyses on ATEs warrant more utilization of methods for distributional effects or multiple-moderators analyses. In addition, we found that there is a dearth of evidence on 1) how Head Start affects health and parental outcomes, 2) Head Start's long-term and distributional effects, and 3) what aspects of Head Start center quality and contextual factors, such as neighborhood and state characteristics, potentially modify the effect of Head Start. Future research should pay deserved attention to under-explored outcomes, moderators, long-term effects, and distributional effects. This would be an essential step towards more effective services and efficient use of resources for Head Start, as well as for the broader field of child development interventions at large.
