# Supplementary material for: Treatment effect heterogeneity in the head start impact study: A systematic review of study characteristics and findings
Source: SSM Popul Health. 2021 Sep 8;16:100916. doi: 10.1016/j.ssmph.2021.100916 (PMC8455360; doi:10.1016/j.ssmph.2021.100916)
Supplement: Multimedia component 1 [file mmc1.docx]

| **Table A.1** Characteristics of the studies included | | | | | | | | |
| --- | --- | --- | --- | --- | --- | --- | --- | --- |
| **Authors (year)** | **Moderator** | **Methods**  **-** main analysis  - causal estimand  - attrition | **Cohort and assessment year** | **Outcomes** | **Quantitative summary for subgroups** - statistically significant proportions of average treatment effect findings  - by cohort, moderator, assessment year, outcome - p<0.05 - outcome (subgroup +, subgroup -, interaction) | **Conclusion  regarding treatment effect heterogeneity** | **Notes** | **Citation** |
| Lipscomb et al. (2013) | Child care type (baseline) - non-parental care | *Main analysis*  - Subgroup analysis  - Path analysis  *Causal estimand*  - ITT (intent-to-treat)  *Attrition*  *­*- weighting | Combined cohort - Year 1 | Cognitive - Pre-Academic Skills  Teacher-reported social-emotional - teacher-child positive relationship, total behavior problems | *Combined cohort* 1. Child care type - HS year: cognitive (+: 1/1), social-emotional (+:1/2) | - Short-term effects were positive for Pre-Academic Skills, teacher-child relationship, and behavioral problems of children with non-parental care at baseline. | - Indirect effects were excluded. | Lipscomb, S.T., Pratt, M.E., Schmitt, S.A., Pears, K.C., Kim, H.K., 2013. School readiness in children living in non-parental care: Impacts of Head Start. J. Appl. Dev. Psychol. 34, 28–37. |
| Gelber & Isen (2013) | Child characteristics - gender  Household/parental characteristics - race/ethnicity - father at home | *Main analysis*  - Subgroup analysis  *Causal estimand*  - TOT (treatment-on-the-treated)  *Attrition*  *­*- weighting  - imputation  - estimate bounds | Combined cohort  - Year 1  - age 4, kindergarten, 1st grade combined | Parenting - parent involvement (a composite measure of multiple parental outcomes) | *Combined cohort* 1. Child characteristics - 1st year: parenting (+: 4/5) - age 4 to 1st grade combined: (+: 2/5) 2. Household/parental characteristics - 1st year: parenting (+: 2/2) - age 4 to 1st grade combined: (+: 1/2) | - The positive effects on parental involvement are much larger for Blacks and Hispanics compared to Whites, and similar across gender, or those with and without a father at home. | - The findings from age 4 to 1st grade were combined so they were not included in the quantitative summary. | Gelber, A., Isen, A., 2013. Children’s schooling and parents’ behavior: Evidence from the Head Start Impact Study. J. Public Econ. 101, 25–38. |
| Bitler, et al. (2014) | Child characteristics - language - cognitive skills at baseline  Household/parental characteristics - race/ethnicity | *Main analysis*  - Quantile regression - Subgroup analysis  *Causal estimand*  - ITT  - TOT  *Attrition*  *­*- weighting | 3-year-old cohort - age 3, age 4, kindergarten, 1st grade | Cognitive  - PPVT, Pre-Academic Skills, Letter-Word Identification, Applied Problems, Spelling | *3-year-old cohort* 1. Child characteristics - age 3: cognitive (+: 4/5) 2. Household/parental characteristics - age 3: cognitive (+: 2/3) | - Short-term effects were positive on cognitive outcomes and the largest at the bottom of the outcome distribution.  - Long-term effects were positive for Spanish speakers, while they faded for the rest. | - IV estimates were included.  - Parent-reported social-emotional and teacher-reported social-emotional outcomes are excluded because the heterogeneous impacts were not assessed. | Bitler, M.P., Hoynes, H.W., Domina, T., 2014. Experimental Evidence on Distributional Effects of Head Start (No. 20434), NBER Working Paper. National Bureau of Economic Research, Inc., Cambridge, MA. |
| Cooper & Lanza (2014) | Child, household/parental characteristics - latent classes  - married, lower risk  - married, dual language, low education  - single, Food Stamps, depression  - single, higher education, full-time  - single, dual language, low education | *Main analysis*  - Latent class analysis  *Causal estimand*  - ITT  *Attrition*  *­*- weighting | 3-year-old cohort - age 4, kindergarten, 1st grade | Cognitive - PPVT, Letter-Word Identification, Applied Problems  Parent-reported social-emotional - total behavior problems, social skills and positive approaches to learning, parent-child positive relationship  Teacher-reported social-emotional - total behavior problems, teacher-child positive relationship | *3-year-old cohort* 1. Child, household/parental characteristics - age 4: cognitive (+: 4/15, interaction: 6/30), social-emotional (+: 4/30, -: 4/30, interaction: 14/60),  - kindergarten: cognitive (-:1/15, interaction: 4/30), social-emotional (+: 3/30. -: 1/30, interaction: 7/60) - 1st grade: cognitive (+:1/15, interaction: 2/30), social-emotional (+: 4/30, -: 1/30, interaction: 11/60) | - Short-term effects on cognitive outcomes were positive for most latent groups, although the long-term effects were nonsignificant.  - The effects on social-emotional outcomes were inconsistent.  - The effects were consistently positive for the Married, ELL, Low Education group, while limited effects were found for the Married, Lower Risk group. |  | Cooper, B.R., Lanza, S.T., 2014. Who benefits most from Head Start? Using latent class moderation to examine differential treatment effects. Child Dev. 85, 2317–2338. |
| Zhai, et al. (2014) | Child care type (counterfactual) - parental care complier - non-parental care complier - center-based care complier - always Head Start | *Main analysis*  - Principal stratification (principal score matching) - Subgroup analysis  *Causal estimand*  - ITT  - TOT  *Attrition*  *­*- weighting | 3-year-old cohort - age 3, age 4, kindergarten, 1st grade  4-year-old cohort - age 4, kindergarten, 1st grade | Cognitive - PPVT, Letter-Word Identification, Applied Problems  Parent-reported social-emotional - social skills and positive approaches to learning, aggressive behaviors, hyperactive behaviors | *3-year-old cohort* 1. Child care type - age 3: cognitive (+: 7/12), social-emotional (+: 3/12) - age 4: cognitive (+: 3/12), social-emotional (+: 4/12) - kindergarten: cognitive (0/12), social-emotional (+: 5/12) - 1st grade: cognitive (+:2/12), social-emotional (+: 2/12)  *4-year-old cohort* 1. Child care type - age 4: cognitive (+: 4/12), social-emotional (+: 1/12) - kindergarten: cognitive (+: 2/12), social-emotional (+: 1/12) - 1st grade: cognitive (+:3/12), social-emotional (+: 2/12) | - The effects were most positive for parental care compliers and non-parental care compliers, while the effects were minimal for center-based care compliers. |  | Zhai, F., Brooks-Gunn, J., Waldfogel, J., 2014. Head Start’s Impact Is Contingent on Alternative Type of Care in Comparison Group. Dev. Psychol. 50, 2572–2586. |
| Miller et al. (2014) | Household/parental characteristics - Preacademic cognitive stimulation | *Main analysis*  - Interaction analysis  *Causal estimand*  - ITT  *Attrition*  *­*- weighting | Combined cohort - Year 1 | Cognitive - PPVT, Letter-Word Identification, Applied Problems | *Combined cohort* 1. Household/parental characteristics - Year 1: cognitive (interaction: 3/6) | - The effects on Applied Problems were positive and the largest for children with low parental preacademic cognitive stimulation. - The effects on PPVT and Letter-Word Identification were positive and the largest for children with moderate parental preacademic cognitive stimulation. |  | Miller, E.B., Farkas, G., Vandell, D.L., Duncan, G.J., 2014. Do the Effects of Head Start Vary by Parental Preacademic Stimulation? Child Dev. 85, 1385–1400. |
| Sabol & Chase-Lansdale (2015) | Household/parental characteristics - parental education level - parental age  - race/ethnicity - country of origin - marital status - number of children - number of adults contribute to income - household income  Neighborhood characteristics: - urbanicity | *Main analysis*  - Subgroup analysis - Interaction analysis  *Causal estimand*  - ITT  - TOT  *Attrition*  *­*- weighting | 3-year-old cohort - age 3, age 4, kindergarten, 1st grade  4-year-old cohort - age 3, age 4, 1st grade | Parental - educational advancement | *3-year-old cohort* 1. Household/parental characteristics - age 3: parental (+: 2/18, interaction: 0/9) - age 4: parental (+: 1/18, interaction: 0/9) - kindergarten: parental (+: 4/18, interaction: 1/9) - 1st grade: parental (+: 2/18, interaction: 1/9) 2. Neighborhood characteristics - age 3: parental (0/2, interaction: 1/1) - age 4: parental (0/2, interaction: 0/1) - kindergarten: parental (+: 1/2, interaction: 0/1) - 1st grade: parental (0/2, interaction: 0/1)  *4-year-old cohort* 1. Household/parental characteristics - age 4: parental (+: 2/18, interaction: 1/9) - kindergarten: parental (0/18, interaction: 1/9) - 1st grade: parental (+: 1/18, interaction: 0/9) 2. Neighborhood characteristics - age 4: parental (0/2, interaction: 0/1) - kindergarten: parental (0/2, interaction: 1/1) - 1st grade: parental (0/2, interaction: 1/1) | - Head Start parents in the 3-year-old cohort increased their educational attainment, and the effects were largest for parents who had at least some college experience at baseline and African American parents. | - Employment outcomes were excluded because heterogeneous impacts were not examined. | Sabol, T.J., Chase-Lansdale, P.L., 2015. The Influence of Low-Income Children’s Participation in Head Start on Their Parents’ Education and Employment. J. Policy Anal. Manag. 34, 136–161. |
| Walters (2015) | Center characteristics - staff with bachelor's degree - staff that have a teaching license - student/staff ratio - full-day service - home visits - High/Scope curriculum - center director experience - center-based care complier proportion  Household/parental characteristics - parental education level - household income  Child characteristics - cognitive skills at baseline | *Main analysis*  - Selection model - Random effect model - Interaction analysis  *Causal estimand*  - TOT  *Attrition*  *­*- weighting | 3-year-old cohort - age 3, age 4, kindergarten, 1st grade  4-year-old cohort - age 4, kindergarten, 1st grade  Combined cohort - Year 1 | Cognitive - a cognitive summary index   Parent-reported social-emotional - a noncognitive summary index | *Combined cohort* 1. Center characteristics - Year 1: cognitive (interaction: 1/8), social-emotional (interaction: 1/8) 2. Household/parental characteristics - Year 1: cognitive (interaction: 1/2), social-emotional (interaction: 0/2) 3. Child characteristics - Year 1: cognitive (interaction: 0/1), social-emotional (interaction: 0/1) | - There was a substantial variation in the effects across the centers. - Centers with full-day service and home visits were more effective, while other characteristics such as High/Scope curriculum, teacher education, and class size did not add to effectiveness. | - Added from reference search  - Treatment effect variation across the Head Start centers | Walters, C.R., 2015. Inputs in the Production of Early Childhood Human Capital: Evidence from Head Start †. Am. Econ. J. Appl. Econ. 7, 76–102. |
| Bloom & Weiland (2015) | Child characteristics - cognitive skills at baseline - dual language - special needs - gender  Household/parental characteristics - race/ethnicity  - home language | *Main analysis*  - Multilevel model - Subgroup analysis  - Interaction analysis  *Causal estimand*  - ITT  - TOT  *Attrition*  *­*- none | Combined cohort - Year 1, Year 3 | Cognitive - PPVT, Letter-Word Identification, Oral Comprehension, Applied Problems  Parent-reported social-emotional - externalizing behavior problems, self-regulation (Leiter Revised) | *Combined cohort* 1. Child characteristics - Year 1: cognitive (+: 30/48, interaction: 8/24), social-emotional (+: 4/16, interaction: 2/8) - Year 3: cognitive (0/12, interaction: 1/6) 2. Household/parental characteristics - Year 1: cognitive (+: 11/20, interaction: 4/8), social-emotional (0/10, interaction: 0/4) | - There was a substantial variation in the effects across centers. - Variance of outcome was reduced for Head Start children. - The effects were largest for dual language learners and Spanish speaking children with low pretest performance on PPVT. | - Treatment effect variation across the Head Start centers  - Treatment effect on variance of outcome | Bloom, H.S., Weiland, C., 2015. Quantifying Variation in Head Start Effects on Young Children’s Cognitive and Socio-Emotional Skills Using Data from the National Head Start Impact Study, MDRC. |
| Pratt, et al. (2015) | Child care type (baseline) - non-parental care | *Main analysis*  - Subgroup analysis  *Causal estimand*  - ITT  *Attrition*  *­*- weighting | Combined cohort - Year 1 | Health - health-related child services (a composite measure)  Parental - preschool-based parent involvement (a composite measure), home-based parental parent involvement (a composite measure), use of physical discipline, parent services (a composite measure) | *Combined cohort* 1. Child care type - age 3: health (+: 1/1), parental (+: 3/6) | - The effects were positive on use of physical discipline and preschool-based parent involvement for children in non-parental care at baseline.  - The effects on home-based parental involvement were nonsignificant. | - Outcomes for home visits receipt and parent services are excluded because they do not belong to main outcomes of interest (cognitive, social-emotional, health, and parental). | Pratt, M.E., Lipscomb, S.T., Schmitt, S.A., 2015. The effect of Head Start on parenting outcomes for children living in non-parental care. J. Child Fam. Stud. 24, 2944–2956. |
| Long (2015) | Household/parental characteristics - marital status - parental education level | *Main analysis*  - Subgroup analysis  *Causal estimand*  - ITT  - TOT  *Attrition*  *­*- weighting | 3-year-old cohort - age 3  4-year-old cohort - age 4 | Parental - employment, full-time job, part-time job, course enrollment, monthly household income, TANF/SSI receipt, Food Stamps/WIC | *3-year-old cohort* 1. Household/parental characteristics - age 3: parental (+: 6/32)  *4-year-old cohort* 1. Household/parental characteristics - age 4: parental (-: 1/32) | - Head Start increased educational course enrollment for single parents, and full-time employment for married parents and less educated parents. - Head Start decreased part-time employment for married parents and full-time employment for more educated parents. |  | Long, C., 2015. Promoting Family Economic Self-Sufficiency: The Impact of Head Start on Maternal Human Capital Investment. Chicago, Illinois. |
| Kline & Walters (2016) | Child care type (counterfactual) - home-based care complier - center-based care complier | *Main analysis*  - Selection model  *Causal estimand*  - ITT  - TOT  *Attrition*  *­*- not reported | Combined cohort - Year 1 | Cognitive - a cognitive composite measure | *Combined cohort* 1. Child care type - Year 1: cognitive (+: 1/2) | - The effects on cognitive outcomes were positive and the largest for home-based care compliers. | - Full model estimates were included.  - Only cohort and assessment year for which heterogeneous effect was explored were reported. | Kline, P., Walters, C.R., 2016. Evaluating Public Programs with Close Substitutes: the Case of Head Start. Q. J. Econ. 131, 1795–1848. |
| Feller et al. (2016) | Child characteristics and child care type (counterfactual) - always Head Start - always center-based care - always home-care care - center-based care complier - home-based care complier -cognitive skills at baseline  - dual language | *Main analysis*  - Principal stratification - Quantile regression  *Causal estimand*  - ITT  - TOT  *Attrition*  *­*- hierarchical Bayesian estimation | 3-year-old cohort - age 3, age 4, kindergarten, 1st grade  4-year-old cohort - age 4, kindergarten, 1st grade  Combined cohort - Year 1 | Cognitive - PPVT | *Combined cohort* 1. Child care type, child characteristics - Year 1: cognitive (+:1/2) 2. Child characteristics, child care type - Year 1: cognitive (+: 4/8)  *3-year-old cohort* 1. Child care type, child characteristics - Year 1: cognitive (+: 1/2) - age 4: cognitive (+ : 1/2) - kindergarten: cognitive (0/2) - 1st grade: cognitive (+: 1/2)  *4-year-old cohort* 1. Child care type, child characteristics - age 4: cognitive (+: 1/2) - kindergarten: cognitive (0/2) - 1st grade: cognitive (+: 1/2) | - The short-term effects on PPVT were positive and strong for home-based compliers. - No effects were found for center-based care compliers. | - Quantile treatment effect | Feller, A., Grindal, T., Miratrix, L., Page, L., 2016. Compared to what? Variation in the impacts of early childhood education by alternative care type. Ann. Appl. Stat. 10, 1245–1285. |
| Miller, et al (2016) | Child characteristics - cognitive skills at baseline - social-emotional measures at baseline - never seen a dentist - suboptimal overall health  Household/parental characteristics - parental depression symptoms - literacy level - economic difficulty | *Main analysis*  - Interaction analysis  *Causal estimand*  - ITT  *Attrition*  *­*- weighting  - imputation | Combined cohort - Year 1 - kindergarten | Cognitive - Pre-Academic Skills  Parent-reported social-emotional - total behavior problems  Teacher-reported social-emotional - total behavior problems | *Combined cohort* 1. Child characteristics - Year 1: cognitive (interaction: 0/7), social-emotional (interaction: 3/7) - kindergarten: cognitive (interaction: 0/7), social-emotional (interaction: 3/14) 2. Household/parental characteristics - Year 1:cognitive (interaction: 0/3), social-emotional (interaction: 0/3) - kindergarten: cognitive (0/3), social-emotional (1/6) 3. Child, household/parental characteristics - Year 1: cognitive (interaction: 0/1), social-emotional (interaction: 0/1) - kindergarten: cognitive (interaction: 0/1), social-emotional (interaction: 1/2) | - The short-term effects on Pre-Academic Skills were not differential by risk factors targeted by Head Start programs. |  | Miller, E.B., Farkas, G., Duncan, G.J., 2016. Does Head Start differentially benefit children with risks targeted by the program’s service model? Early Child. Res. Q. 34, 1–12. |
| McCoy et al. (2016) | Neighborhood characteristics  - urbanicity | *Main analysis*  - Subgroup analysis - Interaction analysis  *Causal estimand*  - ITT  *Attrition*  *­*- none | Combined cohort - Year 1 | Cognitive - PPVT, Letter-Word Identification, Oral Comprehension, Spelling | *Combined cohort* 1. Neighborhood characteristics - Year 1: cognitive (+: 3/6, interaction: 1/4) | - The effects on PPVT were more positive for children in urban centers, while the effects on Oral Comprehension were more positive for children in rural centers. |  | McCoy, D.C., Morris, P.A., Connors, M.C., Gomez, C.J., Yoshikawa, H., 2016. Differential effectiveness of Head Start in urban and rural communities. J. Appl. Dev. Psychol. 43, 29–42. |
| Lee & Lee (2016) | Child care type (baseline) - non-parental care  Child characteristics  - gender - special needs - cognitive skills at large  Household/parental characteristics - parental age - marital status - parental education level - household income - race/ethnicity  - book reading to the child at baseline | *Main analysis*  - Subgroup analysis  *Causal estimand*  - endogenous TOT  *Attrition*  *­*- none | Combined cohort - Year 3 | Parent-reported social-emotional - parent-child positive relationship, social skills and positive approaches to learning  Teacher-reported social-emotional - teacher-child positive relationship, inattentive/hyperactive behaviors, aggressive behaviors  Parental - reading to the child | *Combined cohort* 1. Child care type - Year 3: social-emotional (+:1/5), parental (0/1, interaction: 0/1)) 2. Child characteristics, child care type - Year 3: parental (interaction: 1/3) 3. Household/parental characteristics, child care type - Year 2: social-emotional (interaction: 1/5), parental (interaction: 0/6) | - Among children in foster care at baseline, the effects on parental book reading was larger for children with special needs and smaller for children with low preacademic scores. - For children in foster care at baseline, social-emotional outcomes were improved. | - Added from reference search | Lee, K., Lee, J.-S., 2016. Parental Book Reading and Social-Emotional Outcomes for Head Start Children in Foster Care. Soc. Work Public Health 31, 408–418. |
| Ansari, et al. (2016) | Household/parental characteristics - reading to the child at baseline - preacademic cognitive stimulation - use of physical discipline - parental depressive symptoms | *Main analysis*  - Multiple group analysis - Interaction analysis  *Causal estimand*  - ITT  *Attrition*  *­*- weighting | 3-year-old cohort - age 3  4-year-old cohort - age 4 | Parental - reading to the child, parental cognitive stimulation, use of physical discipline, depressive symptoms | *3-year-old cohort* 1. Household/parental characteristics - age 3: parental (+: 5/14, interaction: 4/30)  *4-year-old cohort* 1. Household/parental characteristics - age 4: parental (+: 3/14, interaction: 2/30) | - The effects on book reading and cognitive stimulation were positive regardless of the parents’ baseline parenting behaviors. - The effects on depressive symptoms and use of physical discipline were positive only those with most depressive symptoms and most frequent use of physical discipline. |  | Ansari, A., Purtell, K.M., Gershoff, E.T., 2016. Parenting Gains in Head Start as a Function of Initial Parenting Skill. J. Marriage Fam. 78, 1195–1207. |
| Lee, et al. (2016) | Child characteristics - special needs - number of special needs - receipt of individualized educational plan  Household/parental characteristics: - race/ethnicity - household income - receipt of SSI | *Main analysis*  - Subgroup analysis - Interaction analysis  *Causal estimand*  - endogenous TOT  *Attrition*  *­*- none | Combined cohort - Year 3 | Parent-reported social-emotional - social skills and positive approaches to learning, total behavioral problems, parent-child positive relationship, social competencies  Teacher-reported social-emotional - teacher-child positive relationship | *Combined cohort* 1. Child characteristics - Year 3: social-emotional (0/5, interaction: 1/15) 2. Child, household/parental characteristics - Year 3: social-emotional (interaction: 2/20) | - Among children with special needs, the effects were positive for those who did not have diagnosed disabilities, did not have an individualized educational plan, lived in higher income household, and were African Americans. |  | Lee, K., Calkins, A., Shin, T.S., 2016. Head start impact on social-emotional outcomes for children with disabilities. Res. Soc. Work Pract. 26, 790–802. |
| Lee & Rispoli (2016) | Child characteristics - special needs - number of special needs - receipt of individualized educational plan | *Main analysis*  - Subgroup analysis - Interaction analysis  *Causal estimand*  - endogenous TOT  *Attrition*  *­*- none | Combined cohort - Year 3 | Cognitive - Oral Comprehension, Applied Problems, Math Reasoning, Quantitative Concepts, Pre-Academic Skills | *Combined cohort* 1. Child characteristics - Year 3: cognitive (0/5, interaction: 3/15) | - The effects on cognitive outcomes were positive for children with special needs. |  | Lee, K., Rispoli, K., 2016. Effects of individualized education programs on cognitive outcomes for children with disabilities in Head Start programs. J. Soc. Serv. Res. 42, 533–547. |
| Lee & Ludington (2016) | Child characteristics - experience of violence or neighborhood crime | *Main analysis*  - Subgroup analysis - Interaction analysis  *Causal estimand*  - endogenous TOT  *Attrition*  *­*- none | Combined cohort - Year 3 | Parent-reported social-emotional - social skills and positive approaches to learning, total behavior problems, parent-child closeness | *Combined cohort* 1. Child characteristics - Year 3: social-emotional (interaction: 2/4) | - The effects on social-emotional outcomes were positive and greater for those who had experienced violence or crime. |  | Lee, K., Ludington, B., 2016. Head start’s impact on socio-emotional outcomes for children who have experienced violence or neighborhood crime. J. Fam. Violence 31, 499–513. |
| Lee (2016) | Child characteristics - gender  Household/parental characteristics - parental age  Child care type (baseline) - non-parental care | *Main analysis*  - Subgroup analysis - Interaction analysis  *Causal estimand*  - endogenous TOT  *Attrition*  *­*- none | Combined cohort - Year 3 | Cognitive - Math Reasoning, Oral Comprehension | *Combined cohort* 1. Child care type - Year 3: cognitive (0/2) 2. Child characteristics, child care type - Year 3: cognitive (+: 1/4, -:1/4, interaction: 1/2) 3. Household/parental characteristics, child care type - Year 3: cognitive (+: 0/4, -:1/4, interaction: 0/2) | - There was no significant effect on cognitive outcomes for those in foster care at baseline.  - Among children in foster care at baseline, the effects on Math Reasoning and Oral Comprehension were greater for girls compared to boys, and the effects on Math Reasoning were greater for children with older caregivers than younger caregivers. |  | Lee, K., 2016. Head Start’s Impact on Cognitive Outcomes for Children in Foster Care. Child Abus. Rev. 25, 128–141. |
| Ding, et al. (2016) | Individual | *Main analysis*  - Randomization-based test for treatment effect variation  *Causal estimand*  - ITT  *Attrition*  *­*- none | Combined cohort  - Year 1 | Cognitive  - PPVT | n/a | - There was significant unexplained treatment effect variation after accounting for all observed covariates. | - Individual treatment effect | Ding, P., Feller, A., Miratrix, L., 2016. Randomization inference for treatment effect variation. J. R. Stat. Soc. Ser. B (Statistical Methodol. 78, 655–671. |
| Miller (2017) | Center characteristics - Spanish instruction  Child characteristics - dual language | *Main analysis*  - Subgroup analysis - Interaction analysis  *Causal estimand*  - ITT  *Attrition*  *­*- weighting | Combined cohort - Year 1 | Cognitive - PPVT, Letter-Word Identification, Applied Problems | *Combined cohort* 1. Child characteristics - Year 1: cognitive (+: 2/3) 2. Child, center characteristics - Year 1: cognitive (+: 1/1, interaction: 1/3) | - Among dual language learners, the effects on PPVT were positive and the largest for those who were instructed in Spanish. |  | Miller, E.B., 2017. Spanish instruction in head start and dual language learners’ academic achievement. J. Appl. Dev. Psychol. 52, 159–169. |
| Chor (2018) | Household/parental characteristics - multigenerational Head Start families | *Main analysis*  - Subgroup analysis - Interaction analysis  *Causal estimand*  - ITT  *Attrition*  *­*- weighting | Combined cohort - Year 1, Year 2, Year 3  - 3rd grade | Cognitive - PPVT, Letter-Word Identification, Applied Problems  Parent-reported social-emotional - social skills and positive approaches to learning, aggressive behaviors, hyperactive behaviors, withdrawn behaviors  Parental - parental cognitive stimulation - family cultural enrichment activities | *Combined cohort* 1. Household/parental characteristics - Year 1: cognitive (+: 5/6, interaction: 1/3), social-emotional (+: 2/8, interaction: 0/4), parental (+: 6/8, interaction: 0/4) - Year 2: cognitive (+: 1/6, interaction: 1/3), social-emotional (+: 1/8, interaction: 1/4), parental (+: 2/8, interaction: 0/4) - Year 3: cognitive (+: 2/6, interaction: 3/3), social-emotional (0/8, interaction: 0/4) - 3rd grade: cognitive (+: 1/6, -: 1/6, interaction: 1/6), social-emotional (+: 2/8, interaction: 1/4) | - The effects on cognitive and social-emotional outcomes were positive and large through 3rd grade for children whose mothers that participated in Head Start in their childhood. |  | Chor, E., 2018. Multigenerational Head Start Participation: An Unexpected Marker of Progress. Child Dev. 89, 264–279. |
| Lee (2019) | Center characteristics - center quality composite score | *Main analysis*  - Subgroup analysis - Interaction analysis  *Causal estimand*  - endogenous TOT  *Attrition*  *­*- imputation | Combined cohort - Year 3 | Cognitive - Oral Comprehension  Teacher-reported social-emotional - positive teacher-child relationship | *Combined cohort* 1. Center characteristics - Year 3: cognitive (interaction: 1/3), social-emotional (interaction: 0/3) | - High quality Head Start was marginally more effective than low quality Head Start for cognitive outcomes, but not social-emotional outcomes. | - Added from reference search | Lee, K., 2019. Impact of Head Start Quality on Children’s Developmental Outcomes. Soc. Work Public Health. |
| Shapiro, et al. (2019) | Child characteristics  - special needs | *Main analysis*  - Subgroup analysis - Interaction analysis  *Causal estimand*  - ITT  *Attrition*  *­*- none | Combined cohort  - Year 1  - 1st grade, 3rd grade | Cognitive  - PPVT, Applied Problems, Letter-Word Identification  Parent-reported social-emotional  - externalizing behavior problems | *Combined cohort* 1. Child characteristics  - Year 1: cognitive (+: 11/18), social-emotional (+: 3/6)  - 1st grade: cognitive (+: 4/18), social-emotional (+: 1/6)  - 3rd grade: cognitive (+: 1/18), social-emotional (0/6) | - Special needs children appeared to benefit less than non-special needs children in short-term, but the findings were sensitive to the definitions of special needs. |  | Shapiro, A., Weiland, C., 2019. What Is in a Definition? The How and When of Special Education Subgroup Analysis in Preschool Evaluations. Educ. Eval. Policy Anal. 41, 145–163. |
| Ding et al. (2019) | Individual | *Main analysis*  - test for systematic variation explained by covariates - treatment effect variation explained by covariates  *Causal estimand*  - ITT  - TOT  *Attrition*  *­*- none | Combined cohort  - Year 1 | Cognitive  - PPVT | n/a | - A substantial proportion of total treatment effect variation were explained by the observed covariates. However, a significant amount remained unexplained even after accounting for all observed covariates and noncompliance. | - Individual treatment effect | Ding, P., Feller, A., Miratrix, L., 2019. Decomposing Treatment Effect Variation. J. Am. Stat. Assoc. 114, 304–317. |
| Lee (2020) | Child care type (baseline) - non-parental care | *Main analysis*  - Subgroup analysis - Interaction analysis  *Causal estimand*  - endogenous TOT  *Attrition*  *­*- none | Combined cohort  - Year 3  - 3rd grade | Cognitive  - Applied Problems, Letter-Word Identification  Parent-reported social-emotional  - social skills and positive approaches to learning, total behavior problems  Health  - dental care treatment, medical care for injury | *Combined cohort*  1. Child care type  - Year 3: cognitive (0/4), social-emotional (0/4), health (+: 1/2)  - 3rd grade: cognitive (0/2), social-emotional (0/2), health +: 1/2, -:1/2) | - Head Start increased access to dental care and decreased health care for injury. | - Health care for injury were reduced for Head Start children but this may be due to lower probability of being injured, rather than less access to health care. | Lee, K., 2020. Long-term Head Start Impact on developmental outcomes for children in foster care. Child Abus. Negl. 101. |

| **Table A.2** Frequency of outcomes | | | | |
| --- | --- | --- | --- | --- |
|  | **Outcome** | **# of studies** | **Proportion among all studies within outcome category (%)^1^** | **Proportion among all studies (%)^2^** |
| Cognitive | - Applied Problems | 16 | 80 | 57 |
|  | - Letter-Word Identification | 15 | 75 | 54 |
|  | - PPVT | 14 | 70 | 50 |
|  | - Spelling | 7 | 35 | 25 |
|  | - Oral Comprehension | 6 | 30 | 21 |
|  | - Quantitative Concepts | 3 | 15 | 11 |
|  | - Color identification, CTOPPP Elision, Word Attack, Draw-a-Design Task, Letter Naming, Counting Bears, Leiter Revised (self-regulation) | 1 | 5 | 4 |
|  | - Passage comprehension, Writing Samples, ECLS-K Reading Assessment, Calculation, teacher report on school performance (language and literacy ability, math ability, social studies and science ability, school accomplishments), parent report on school performance (promotion, Emergent Literacy Scale), Print and Story Concept, Writing Name Task, CTOPPP Print Awareness | 0 | 0 | 0 |
|  | **Total # of official-report outcomes = 26** |  |  |  |
|  |  |  |  |  |
| Social-emotional | *Parent-reported outcomes* | 11 | 85 | 39 |
|  | - Aggressive behaviors, hyperactive behaviors | 10 | 77 | 36 |
|  | - Social skills and positive approaches to learning | 8 | 62 | 29 |
|  | - Withdrawn behaviors | 7 | 54 | 25 |
|  | - Closeness | 5 | 38 | 18 |
|  | - Conflict | 4 | 31 | 14 |
|  | - Social competencies | 1 | 8 | 4 |
|  | **Total # of official-report outcomes = 7** |  |  |  |
|  |  |  |  |  |
|  | *Teacher-reported outcomes* | 6 | 46 | 22 |
|  | - Aggressive behaviors, inattentive/hyperactive behaviors, closeness, conflict | 4 | 31 | 15 |
|  | - Oppositional behaviors | 2 | 15 | 7 |
|  | - Withdrawn-low energy, socially reticent behaviors | 1 | 8 | 4 |
|  | - Social competencies, emotional symptoms scale, conduct problems scale, hyperactivity scale, peer problem scale, prosocial scale, total difficulties score, problems with structured learning, problems with peer interaction, problems with teacher interaction | 0 | 0 | 0 |
|  | **Total # of official-report outcomes = 17** |  |  |  |
|  |  |  |  |  |
|  | *Child-reported outcomes* | 0 | 0 | 0 |
|  | - Internalizing, externalizing, peer relations, school experience | 0 | 0 | 0 |
|  | **Total # of official-report outcomes = 4** |  |  |  |
|  |  |  |  |  |
| Health | - Dental care receipt | 2 | 100 | 7 |
|  | - Medical care for an injury in the last month | 1 | 50 | 4 |
|  | - Access to health insurance, child's health, need for ongoing medical care | 0 | 0 | 0 |
|  | *non-official-report outcomes^3^* | *1* | *50* | *4* |
|  | - *hearing/vision screening, access to regular medical check-up* | *1* | *50* | *4* |
|  | **Total # official-report of outcomes = 5** |  |  |  |
|  |  |  |  |  |
| Parenting | - Reading to the child | 3 | 50 | 11 |
|  | - Use of physical discipline, family cultural enrichment activities | 2 | 33 | 7 |
|  | - Use of time out, parental safety practices, parenting styles, parent participation in school activities, school contact/communication, school as a supportive environment, effect of parenting on parent's life, perception of school services, general activities with child, time spent with child | 0 | 0 | 0 |
|  | *non-official-report outcomes* | *3* | *50* | *11* |
|  | - *cognitive stimulation for the child,*  *parent involvement (composite measure)* | *2* | *33* | *7* |
|  | - *educational advancement, depressive symptoms, employment, full-time job, part-time job, course enrollment, monthly household income, TANF/SSI receipt, Food Stamps/WIC receipt, help with housing, help with utilities, job training and employment assistance, substance abuse treatment or counseling, family counselling or mental health services, help with family violence, foster care payments, home visits* | *1* | *17* | *4* |
|  | **Total # of official-report outcomes = 13** |  |  |  |
| ^1, 2^the sum of the proportions may exceed 100 percent because categorization of the studies is not mutually exclusive.  ^3^ total number of official-report of outcomes does not include non-official-report outcomes. | | | | |

| **Table A.3** Frequency of moderators | | | | |
| --- | --- | --- | --- | --- |
|  | **Moderator** | **# of studies** | **Proportion among all studies within moderator category (%)^1^** | **Proportion among all studies (%)^2^** |
| Child characteristics | - baseline cognitive measures | 6 | 43 | 21 |
|  | - language, special needs | 5 | 36 | 18 |
|  | - gender | 4 | 29 | 14 |
|  | - receipt of individualized educational plan | 2 | 14 | 7 |
|  | - baseline social-emotional measures, baseline health measures, experience of violence or neighborhood crime | 1 | 7 | 4 |
|  |  |  |  |  |
| Household/  parental characteristics | - race/ethnicity | 6 | 46 | 21 |
|  | - parental education level, marital status | 5 | 38 | 18 |
|  | - household income | 4 | 31 | 14 |
|  | - parental age, parental depression symptoms | 3 | 23 | 11 |
|  | - preacademic cognitive stimulation, reading to the child | 2 | 15 | 7 |
|  | - home language, household risk, country of origin, number of children, number of adults contributing to income, use of Food Stamp, use of physical discipline, parental literacy, economic difficulty, receipt of Social Security Income, multigenerational HS family | 1 | 8 | 4 |
|  |  |  |  |  |
| Neighborhood characteristics | - household urbanicity, Head Start center urbanicity | 1 | 50 | 4 |
|  |  |  |  |  |
| Center characteristics | - teacher/care provider education, student:staff ratio, number of home visits per year | 2 | 50 | 7 |
|  | - teacher/care provider training, parent involvement, full-day service, High/Scope curriculum, center director's experience, center-based care complier share, Spanish instruction, ECERS-R or Family Day Care Rating Scale, Arnett Caregiver Interpretation Scale, literacy activities, mathematics activities, other instructional activities, program services to children and families | 1 | 25 | 4 |
|  |  |  |  |  |
| Child care type | - baseline care type, non-parental care | 5 | 63 | 18 |
|  | - counterfactual care type | 3 | 38 | 11 |
| ^1, 2^ the sum of the proportions may exceed 100 percent because categorization of the studies is not mutually exclusive. | | | | |

**Table A.4** Frequency of assessment years studied by targeted cohort

| **Targeted Cohort** | **Assessment year** | **# of studies** | **Proportion among all studies within cohort (%)^1^** | **Proportion among all studies (%)^2^** |
| --- | --- | --- | --- | --- |
| 3-year-old | - age 3 = Year 1 | 7 | 88 | 25 |
|  | - age 4 = Year 2 | 6 | 75 | 21 |
|  | - kindergarten = Year 3 | 6 | 75 | 21 |
|  | - 1st grade = Year 4 | 6 | 75 | 21 |
|  | - 3rd grade = Year 5 | 0 | 0 | 0 |
|  |  |  |  |  |
| 4-year-old | - age 4 = Year 1 | 6 | 100 | 21 |
|  | - kindergarten = Year 2 | 4 | 67 | 14 |
|  | - 1st grade = Year 3 | 4 | 67 | 14 |
|  | - 3rd grade = Year 4 | 0 | 0 | 0 |
|  |  |  |  |  |
| Combined | Time since Head Start | | | |
|  | - Year 1 | 15 | 68 | 54 |
|  | - Year 2 | 1 | 4 | 4 |
|  | - Year 3 | 9 | 39 | 32 |
|  | Academic year | | | |
|  | - age 4 | 1 | 4 | 4 |
|  | - kindergarten | 2 | 9 | 7 |
|  | - 1st grade | 2 | 9 | 7 |
|  | - 3rd grade | 3 | 13 | 11 |
| ^1, 2^ the sum of the proportions may exceed 100 percent because categorization of the studies is not mutually exclusive. | | | | |
